# Characterization of fungal communities in Guánica State Forest caves utilizing internal transcribed spacer sequencing

**DOI:** 10.1128/mra.00647-26

**Published:** 2026-07-10

**Authors:** Peter A. Camacho-Nazario, Camila M. Martínez-Llaurador, Isabel Aguirre-Ortiz, Francisco J. Román-Soto, Naomy M. Chaparro-Mata, Josué Rodríguez-Ramos, Ángel M. Nieves-Rivera, Miguel J. Martínez-González, Miguel Canals-Mora, Carlos Rios-Velazquez

**Affiliations:** 1 Microbial Biotechnology and Bioprospecting Laboratory, Biology Department, University of Puerto Rico 585475, Mayagüez, Puerto Rico; 2 Industrial Biotechnology Program, University of Puerto Rico 16146, Mayagüez, Puerto Rico; 3 Biological Sciences Division, Pacific Northwest National Laboratory 536904, Richland, Washington, USA; 4 Grupo Espeleológico del Oeste, Inc. (GEO), Mayagüez, Puerto Rico; Nanchang University, Nanchang, Jiangxi, China

**Keywords:** Guánica State Forest, Puerto Rico, cave microbiology, taxonomic ITS profile, diversity

## Abstract

Caves are underground, extreme ecosystems harboring diverse microbial communities. This study employed internal transcribed spacer sequencing across four caves and one cave pond-mud sample in the Guánica State Forest to investigate fungal communities. This diversity profile offers a foundation for investigations of fungal populations thriving in Puerto Rican cave systems.

## ANNOUNCEMENT

**Fig 1:**
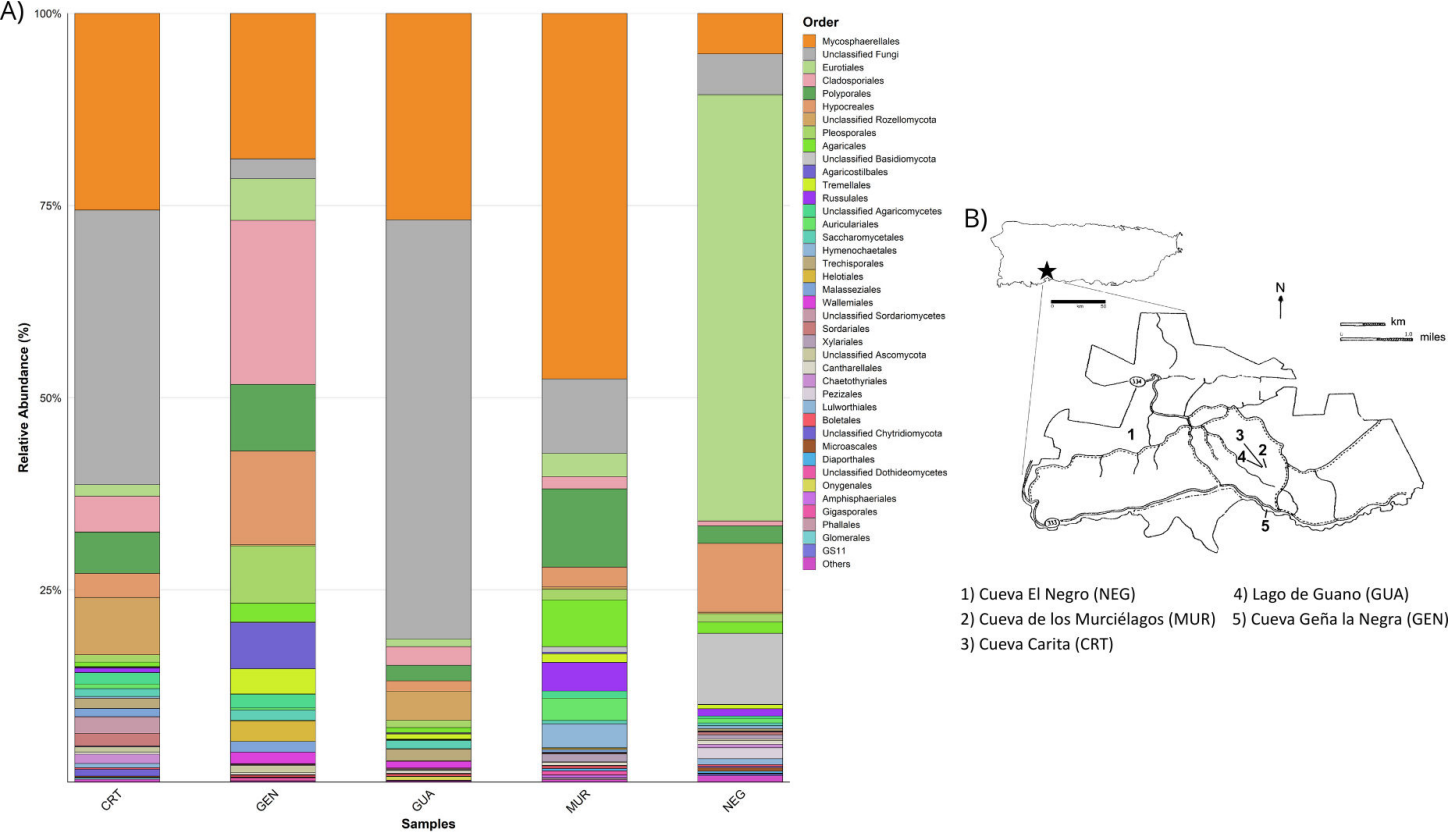
Relative abundance of fungal communities at the order level among four Puerto Rican caves and one pond. (**A**) Taxa bar plots showing the relative abundance of fungal orders in samples. (**B**) Map of Guánica State Forest indicating the locations of the samples. The map was generated by Ángel M. Nieves-Rivera.

Caves are oligotrophic, dark ecosystems with fluctuating temperatures (1, 2). Despite this, they harbor diverse microbial communities involved in nutrient cycling and the production of compounds with biomedical potential (2). Fungi contribute to decomposition, mineral weathering, and a bioprospecting source of novel bioactive compounds (3, 4). Southern Puerto Rican caves, particularly those in Guánica State Forest, remain microbiologically underexplored. The forest’s largest cave system, Cueva de Los Murciélagos, contains interconnected chambers and multiple entrances (5). One of these entrances is Cueva Carita, where bat guano enriches pond mud and may support distinct fungal communities (5). To characterize this diversity, taxonomic profiling was performed on these microbial communities. Four caves and one pond mud within the Guánica State Forest were sampled (Fig. 1; Table 1). Thirty grams of superficial bat guano-enriched soil and pond mud were collected from the caves and stored in sterile Whirl-Pak bags and stored at 4°C until DNA extraction. Genomic DNA was extracted using 10 g of each sample suspended in 20 mL of Z-buffer containing 0.02 g of lysozyme. Cells were disrupted by repeated freeze-thaw cycles, followed by lysis with 2.25 mL of sodium dodecyl sulfate and 1.5 mL of guanidinium isothiocyanate at 65°C for 2 h. Lysates were clarified by centrifugation and extracted with an equal volume of chloroform, and the aqueous phase was precipitated with one volume of isopropanol at −20°C overnight. DNA pellets were centrifuged, washed with 70% ethanol, air dried, resuspended in sterile water, and stored at −80°C (6–8). DNA fragments up to 25 kb were separated by gel electrophoresis and purified with β-agarase I according to the manufacturer’s protocol (9). Purified DNA was sent to Molecular Research DNA Laboratory (MR. DNA, Shallowater, TX, USA) for internal transcribed spacer sequencing using the bTEFAP Illumina 20k in-house ITS1-2 diversity assay (10). The ITS1 region was amplified with ITS1 (5′-TCCGTAGGTGAACCTGCGG-3′) and ITS2 reverse (5′-GCTGCGTTCTTCATCGATGC-3′) using the HotStarTaq Plus Master Mix Kit (Qiagen, USA) (11). PCR consisted of an initial denaturation at 95°C for 5 min, 30 cycles of denaturation at 95°C for 30 s, annealing at 53°C for 40 s, and extension at 72°C for 1 min, with a final extension at 72°C for 10 min. Sequencing was performed on an Illumina MiSeq instrument using 2 × 300 bp paired-end sequencing. Raw sequence data were imported into QIIME 2 version 2026.1 within a Python 3.10.14 environment as demultiplexed paired-end reads and subsequently trimmed with ITSxpress version 2.1.4 (12). Denoising was then performed with DADA2 without additional trimming or truncation (13). Default parameters were used for all bioinformatic software unless otherwise specified. Representative sequences were taxonomically classified using the UNITE version 10.0 dynamic fungi classifier released on 19 February 2025 for QIIME 2 version 2026.1 (14). Across MUR, CRT, GEN, NEG, and GUA, more than 2.0 million non-chimeric reads were retained, averaging 79.21% retention (Table 1). A total of 1,720 ASVs were identified. Unclassified fungi constituted 21.56% of reads. Across classified groups, Mycosphaerellales (24.83%), Eurotiales (13.27%), Cladosporiales (6.13%), and Polyporales (5.70%) were the most abundant classified orders revealing substantial taxonomic variation among sites (Fig. 1).

**TABLE 1:** Sample ID, sample details, and DADA2 analysis results

Sampled caves	Sample ID	Coordinates	Collection date	Raw input reads	Percentage of input passed filter	Denoised	Non-chimeric reads	Percentage of non-chimeric reads
Cueva de los Murciélagos	MUR	17° 57′34.5″ N, 66° 50′58.6″ W	17/05/2025	475,324	97.5	459,975	321,463	69.63
Cueva Carita	CRT	17° 57′16″ N, 66° 50′54″ W	17/05/2025	523,073	94.72	493,996	397,131	75.92
Cueva Geña La Negra	GEN	17° 57′19″ N, 66° 51′10″ W	28/06/2025	590,545	95.87	564,651	480,654	81.39
Cueva El Negro	NEG	17° 58′11″ N, 66° 52′35″ W	28/06/2025	676,466	96.06	647,747	605,844	89.56
Lago de Guano	GUA	17° 57′37″ N, 66° 51′08″ W	17/05/2025	362,443	96.76	348,842	276,427	76.27

## Data Availability

The raw data from this project have been deposited in the National Center for Biotechnology Information (NCBI) under the BioProject accession number PRJNA1457880. The samples were distributed as follows: Cueva Carita (CRT) (SRA accession number SRX33109457), Cueva Gena La Negra (GEN) (SRA accession number SRX33109458), Lago de Guano (GUA) (SRA accession number SRX33109460), Cueva de los Murciélagos (MUR) (SRA accession number SRX33109456), and Cueva El Negro (NEG) (SRA accession number SRX33109459).
